# Screening of Moderately Halophilic Bacteria Producing Ectoine Resulting in the Selection of *Virgibacillus salarius* BHTA19

**DOI:** 10.17113/ftb.63.03.25.8727

**Published:** 2025-08-31

**Authors:** Martyna Leszczewicz, Natalia Broncel, Oliwia Frączak, Tomasz Kapela, Krzysztof Makowski

**Affiliations:** Biotechnika Poland Ltd., Tymienieckiego 25, 90-350 Lodz, Poland

**Keywords:** halophilic bacteria, ectoine, hydroxyectoine, *Virgibacillus salarius*

## Abstract

**Research background:**

Ectoine is a desirable molecule with high application potential, particularly in the cosmetics and pharmaceutical industries. The current production method uses microorganisms that require high salinity. Therefore, purification of the product is expensive, complex and requires appropriate equipment. To overcome these obstacles, we were looking for new moderately halophilic, ectoine-producing bacteria.

**Experimental approach:**

The bacteria were isolated from high-salinity environments: in the vicinity of Tyrawa spring, in Złockie near the Na Mokradłach spring and in Rajcza, all in Poland. Their ability to biosynthesise ectoine and additionally hydroxyectoine in a 10 % premixed seawater environment was assessed semiquantitatively using mass spectrometry (MS). The growth of the bacteria was also compared under these conditions. The most promising strains were then identified based on 16S rDNA sequence and their morphological, biochemical and physiological properties were described. The ectoine was biosynthesised based on the collected data and the preferences of individual strains. The concentrations of the final product were determined by HPLC. After the screening process, the most suitable strain was identified.

**Results and conclusions:**

Fifty-six bacterial strains were isolated. Most strains produced insignificant amounts of ectoine or hydroxyectoine in the presence of 10 % salt. However, ten strains, all isolated from the Tyrawa spring, showed promising properties and were used in further studies. Based on the 16S rDNA sequence, four were identified as *Halobacillus* sp., two as *Virgibacillus* sp. and one as *Bacillus* sp., *Pseudalkalibacillus* sp., *Salimicrobium* sp. and *Thalassobacillus* sp. The basic biochemical and physiological properties as well as the ability to grow in the presence of NaCl, KCl, (NH_4_)_2_SO_4_ and MgSO_4_ were described. Ectoine was biosynthesised following the best parameters estimated for each strain. Based on the results, *Virgibacillus salarius* BHTA19 was identified as a new potential producer of ectoine.

**Novelty and scientific contribution:**

We isolated a promising ectoine producer, moderately halophilic bacterium - *Virgibacillus salarius* BHTA19. BHTA19 is a wild-type strain that produces significant amounts of ectoine in environments with moderate salt concentrations. It has great potential and the possibility of industrial application.

## INTRODUCTION

Living organisms that have colonised planet Earth for billions of years have evolved mechanisms and strategies that allow them to inhabit environments that are potentially harmful or lethal from an anthropocentric point of view. One of the groups of organisms that have had the longest evolution time to adapt to harsh habitats is bacteria. These inhabit the most deadly and unfriendly environments for humans and are in general called extremophiles. Depending on the factor that limits the growth of the microbes, they are categorised as thermophiles (elevated temperature), psychrophiles (low temperature), acidophiles (low pH), alkaliphiles (high pH), radiophiles (high radiation resistance), piezophiles and barophiles (high pressure), xerophiles (low water activity) and polyextremophiles (tolerating or preferring more than one extreme factor) (*1*-*3*). Another interesting group of extremophiles are the halophiles. The bacteria grow and proliferate in environments with high salinity, *e.g.* salt mines, ponds and springs or salt deposits on rocks or buildings (*4*, *5*). Colonization and the ability to proliferate in environments with extreme osmotic pressure is a major challenge for bacteria. Microorganisms have a low cell volume to surface ratio, which can lead to easier loss of intracellular water and consequently make it difficult for them to effectively control the osmotic balance (*6*).

To survive, halophiles have developed specific intracellular mechanisms that allow them to retain intracellular water. In general, they have two main mechanisms of action. The first is called the "salt-in strategy" and is based on the accumulation of salts within the cell, the concentration of which balances external osmotic pressure and prevents leakage of water. This is common in archaea and certain *Halobacteria* (*7*). The second method, which is more common among procaryotes, is much more complicated and is based on the synthesis of small organic molecules within the cell called compatible solutes or osmoprotectants. These molecules are produced in the cells in high concentrations without affecting central metabolism and they belong mainly to the polyols and carbohydrate derivatives. These compounds are commonly found in bacterial and plant kingdoms, including glycerol, *myo*-inositol, glucosylglycerol and trehalose (*8*).

In terms of potential commercial application, two hydroxyproline derivatives, osmoprotectants – ectoine and its hydroxylated derivative – hydroxyectoine, are worth mentioning. Both bind water molecules strongly and are found in bacterial cells. Their concentration depends on the type of bacteria and the environmental conditions. Ectoine concentration can increase from a few millimoles per litre to up to 100 mM in response to stress like high salt, drought or temperature changes (*1*).

Apart from their osmoprotective properties, they also act as antifreezing agents, UV filters, soothing and anti-inflammatory molecules (*9*). These properties make both molecules incredibly attractive components for the cosmetic and food industries. Due to their low irritability, they are also widely used in pharmaceutical products, including high-purity drugs like eye or nose drops (*10*).

The primary aims of the research were to isolate a wild-type bacterial strain that synthesises ectoine at low or moderate salt concentrations and to carry out preliminary work to find cost-effective and simple growth media.

## MATERIALS AND METHODS

### Isolation of halophilic bacteria

Halophilic bacteria were isolated from highly saline environments: in the vicinity of Tyrawa spring, in Złockie near Na Mokradłach spring and in Rajcza, all in Poland. The samples, which contained a combination of bottom sediments and water, were kept at 4 °C until use. For isolation, 10 g of well-mixed sample were added to 100 mL of buffered peptone water (BTL, Lodz, Poland), then the dilutions were shaken for 30 min at 30 °C and 150 rpm. Halophilic bacteria were screened on nutrient agar (BTL) with the addition of 100 g/L of premixed salts Instant Ocean (Aquarium Systems, Sarrebourg, France). The samples were incubated at 30 °C for one to two weeks. The individual colonies were then harvested and streaked onto Petri dishes with the previously used medium. After 24 h of incubation at 30 °C, the purity of the culture was examined after Gram staining under the light microscope at 1000× magnification (BX63; Olympus, Tokyo, Japan). When the purity of the culture was confirmed, the isolated bacteria were kept frozen at -80 °C in glycerol stocks. If the isolated bacteria needed further purification, they were streaked again, incubated and observed under the microscope.

### Recovery of bacteria from the glycerol stocks

The cells collected from the glycerol stock were inoculated onto nutrient agar (BTL) with 100 g/L of premixed salts Instant Ocean (Aquarium Systems) and incubated for 24 h at 30 °C.

### Characterisation of bacteria morphology

Microscopic characteristics of the bacterial cells were analysed in fresh cultures obtained directly after the recovery of the bacteria from glycerol stocks. Cell morphology was examined after Gram staining at 1000× magnification using a light microscope (BX63; Olympus).

### Determination of oxidase, l-alanine aminopeptidase and catalase activity

Enzymatic activity was evaluated after recovery of the bacteria from the glycerol stock. The cytochrome oxidase and l-alanine aminopeptidase activity were detected using Bactident® Oxidase and Bactident® Aminopeptidase (both from Merck, Darmstadt, Germany) test strips. The analyses were carried out according to the manufacturer's instructions. Catalase activity was determined by placing the bacterial biomass on a microscope slide and applying a drop of 5 % hydrogen peroxide (Chempur, Piekary Śląskie, Poland). The activity was analysed by gas formation. The presence of catalase was confirmed by the appearance of gas bubbles.

### Bacterial cultivation and ectoine biosynthesis

Cells were recovered from glycerol stock and one loop of the biomass was added to 50 mL of inoculation medium with nutrient broth (BTL) and 100 g/L seawater (Instant Ocean, Aquarium Systems). The bacteria were incubated overnight. Then, 20 mL of the inoculum were transferred to 200 mL of nutrient broth (BTL) and either 100 or 150 g/L seawater was added. The fermentations were conducted at 150 rpm and 30 °C in a shaking incubator (Multitron; Infors HT, Basel, Switzerland).

### Effect of various salt amounts on bacterial growth

Bacteria were recovered from glycerol stock as previously described. The cells were grown in 50 mL medium with nutrient broth (BTL) and 100 g/L seawater (Instant Ocean, Aquarium Systems) at 150 rpm and 30 °C in a shaking incubator (Multitron; Infors HT) and then used as an inoculum. Tubes with 9 mL of the tested media were then inoculated with 1 mL of the culture. A total of 21 types of media were prepared. One contained: 1 g/L beef extract (BTL), 2 g/L yeast extract and 5 g/L peptone (both from VWR, Radnor, PA, USA). The other media also contained one of the following inorganic salts: Instant Ocean (Aquarium Systems), NaCl, MgSO_4_·7H_2_O, (NH_4_)_2_SO_4_ (all from Chempur) or KCl (VWR). The bacterial growth was analysed with five different amounts of each salt: 5, 10, 15, 20 and 25 %. The strains were incubated at 30 °C. The study was carried out for 48 h. Turbidity was measured with a densitometer (DEN-1B; Biosan, Riga, Latvia) to determine the limiting growth parameters. The results were expressed on the McFarland scale. The changes in turbidity were then calculated using the following equation:


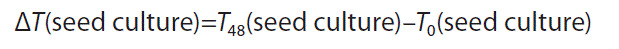
 /1/

where Δ*T* is the change in turbidity, *T*_0_ is the initial turbidity and *T*_48_ is the turbidity after 48 h of incubation. Based on the results, the cardinal salt amounts were determined.

### Evaluation of bacterial growth at different temperatures and pH

The test was performed analogously to the evaluation of the effects of different salt amounts. Each tube contained 9 mL of nutrient broth (BTL) supplemented with 100 g/L of premixed salts from Instant Ocean (Aquarium Systems). This time, the pH of the medium in each tube was different and was adjusted to 3, 4, 5, 6, 7, 8 or 9. The cultures were incubated at 30 °C for 48 h. Additionally, the turbidity changes at pH=7 were also measured after incubation at 10, 15, 20, 25, 37 and 44 °C. To determine the limits of growth as well as the optimum temperature and pH, the increase in turbidity was estimated according to Eq. 1.

### Molecular identification of selected bacteria

Genomic DNA was extracted using NucleoSpin® Microbial DNA kit (Macherey-Nagel GmbH & Co. KG, Düren, Germany) according to the manufacturer’s recommendations. The concentration and purity of the isolated DNA were determined using spectrophotometry (Nanovue Plus; Biochrom, Cambridge, UK). Universal bacterial primer sets 27F: 5’-AGAGTTTGATCCTGGCTCAG-3’ and 1492R: 5’-TACGGTACCTTGTTACGACTT-3’ were used for the amplification of the 16S rRNA genes. The polymerase chain reaction (PCR) master mix mixture contained 4 µL genomic DNA, 1 µL of each primer (concentration 10 µM), 25 µL PrimeSTAR® Max DNA Polymerase (Takara, Kyoto, Japan) and water, so that the final volume was 50 µL. The PCR was carried out in the Agilent SureCycler 8800 (Agilent Technologies, Santa Clara, CA, USA) after 30 cycles under the following conditions: 10 s initial denaturation at 98 °C, 30 cycles of denaturation (10 s at 98 °C), annealing (10 s at 55 °C) and extension (10 s at 72 °C). The PCR products were sequenced by Genomed Inc. (Genomed, Warsaw, Poland) using the Sanger sequencing method.

### Determination of cell growth

The changes in the absorbance values were measured at *λ*=600 nm using Shimadzu UV-1800 UV/Visible scanning spectrophotometer (Shimadzu, Kyoto, Japan). To dissolve the salts present in the medium, the samples were first diluted ten times with 10 % acetic acid (Chempur) and then with water.

### Extraction of ectoine and hydroxyectoine

A volume of 200 mL of broth was centrifuged at 3220×*g* (centrifuge 5810 R; Eppendorf, Hamburg, Germany) and 4 °C for 30 min and then the pellets were suspended in 80 % ethanol (Avantor, Gliwice, Poland). Extraction was carried out at 200 rpm and 25 °C on a shaking incubator (Infors HT). Then, ethanol suspension was centrifuged at 3220×*g* and 4 °C for 30 min and the supernatant was placed in a vacuum dryer at 60 °C and 30 kPa (vacuum oven VO500; Memmert, Schwabach, Germany) until no further change in mass occurred. The dried extract was used for further analysis.

### MS determination of ectoine and hydroxyectoine

The presence of ectoine and hydroxyectoine was confirmed by comparing the mass spectra of the samples with the standards. The content of ectoine and hydroxyectoine was determined semiquantitatively based on the number of mass detector counts of the measured *m*/*z* values (ion intensity), which is proportional to the analyte concentration. However, the ion intensity can be influenced by the matrix effect, which was not investigated in this case. High-resolution mass spectra were recorded on the mass spectrometer Impact HD UHR-QqTOF (Bruker, Billerica, MA, USA). The ESI (+) ion source was operated at a dry temperature of 200 °C, a needle voltage of 4.5 kV, with dry gas flow set up at 6 L/min and nebulizer gas pressure of 200 kPa. The mass analyser operated at a hexapole RF of 60 Vpp, collision RF of 500 Vpp, transfer time of 60 μs and pre-pulse storage of 5 μs. The scan range in MS mode was set up from 50 to 1000 *m*/*z* and the sample time was 1 s. The collision energy for qualitative analysis was set up at 7 eV. The spectrometer was internally calibrated with a sodium formate cluster calibrant before each run, according to the procedure specified by the manufacturer. Ectoine (97.4 % HPLC) and hydroxyectoine (98.1 % HPLC) standards were purchased from Merck and Sigma-Aldrich, Merck (Saint Louis, MO, USA), respectively. The water of 100 % HPLC grade was obtained by double distillation and purification using a HYDROLAB HLP20 water purification system (Hydrolab, Straszyn, Poland). To prepare the standard solutions, 51.4 mg ectoine standard and 21.2 mg hydroxyectoine standard in 10 mL volumetric flasks were weighed on an analytical balance, dissolved in water and made up to a final volume of 10 mL. The solutions were diluted with water to a concentration of 50 µg/mL. To prepare the sample solutions, about 20 mg of each sample was weighed and dissolved in water to a concentration of 20 mg/mL. A small amount of the prepared sample solutions was diluted fourfold to a concentration of 5 mg/mL. All solutions were filtered through 0.2-µm PTFE syringe filters and injected into the mass spectrometer using a syringe pump (180 µL/h).

### HPLC determination of ectoine and hydroxyectoine

 The concentrations of ectoine and hydroxyectoine were determined relative to external standards using the UltiMate 3000 UHPLC system coupled with UV-VIS detection (Thermo Scientific, Thermo Fisher Scientific, Waltham, MA, USA) and an Ultra AQ C18 column 100 mm×2.1 mm×3 µm (Restek, Bellefonte, PA, USA). The water of 100 % HPLC grade was used as a mobile phase. All chromatographic separations were performed at 30 °C with a flow rate of 0.08 mL/min and a run time of 10 min. The detection was selected at 210 nm. The standard solutions prepared according to the procedure described above were diluted to a concentration of 10 µL/mL. To prepare the sample solutions, 10 mg of each sample was weighed in a 1-mL volumetric flask on an analytical balance, dissolved in water, filled to a final volume of 1 mL and diluted 100 times with water. All solutions were filtered through 0.2-µm syringe PTFE filters and transferred to a chromatographic vial.

### Data analysis

Calculations, statistical analyses and graphs were prepared using Microsoft Excel 2010 (*11*). The sequences were analysed with open-source software Chromas and Decipher, then aligned and compared with sequences deposited in GenBank (*12*-*14*). The otofControl and Compass Data Analysis software were used to record and analyse mass spectra (*15*, *16*). HPLC data were collected and analysed using Chromeleon software (*17*).

## RESULTS AND DISCUSSION

We isolated 56 bacterial strains that can grow in the presence of a 10 % salt mixture. Fifteen originated from the vicinity of Tyrawa spring (BHTA), 30 from Złockie in the vicinity of Na Mokradłach spring (BHMM) and 11 from Rajcza (BHEGO).

**Fig. 1:**
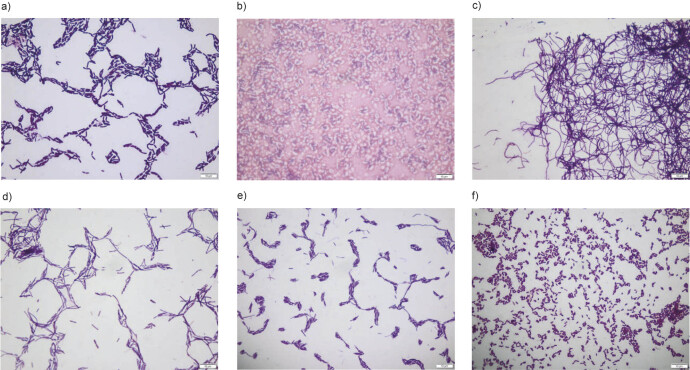
Examples of microscopic observations of isolated bacteria at 1000× magnification: a) BHMME1, b) BHMME8, c) BHTA12, d) BHTA19, e) BHEGO1 and f) BHEGO3

Most bacterial strains were Gram-positive and rod-shaped. BHTA20 and BHTA24 were the only strains classified as cocci. Nevertheless, microscopic observations confirmed that we obtained a diversified set of strains. The morphological differences of the isolates are shown in Fig. 1.

**Fig. 2:**
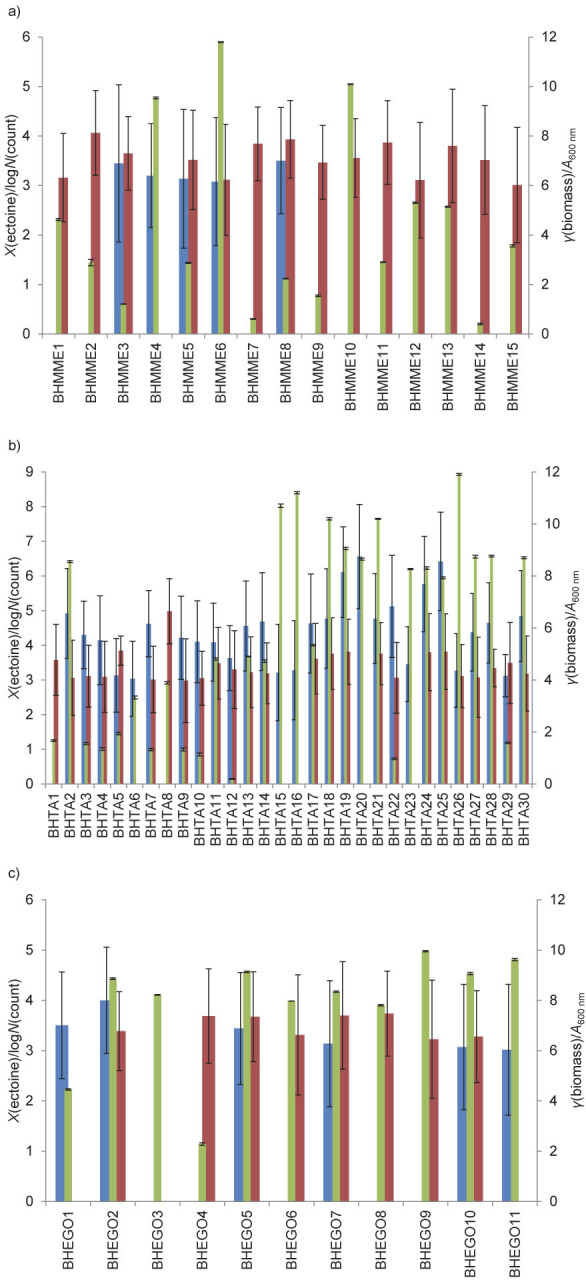
Comparison of the absorbance values at *λ*=600 nm and the LC-MS estimation of the amount of ectoine and hydroxectoine in the bacterial cultures originating from: a) Złockie in the vicinity of Na Mokradłach spring (BHMM), b) the vicinity of Tyrawa spring (BHTA) and c) Rajcza (BHEGO), all in Poland. Blue and red=ectoine and hydroxyectoine number of mass detector counts, respectively, green=absorbance

The more diverse the collection of microorganisms obtained before the screening, the higher the probability of effective selection. Significant morphological differences between the isolated strains suggested positive screening results, *i.e.* the isolation of ectoine producer. The isolated bacteria were grown in a nutrient-rich medium containing 10 % seawater. The presence of ectoine and its derivative hydroxyectoine was then determined semiquantitatively using the LC-MS method. The common logarithm of the number of mass detector counts was calculated for easier presentation of the results (Fig. 2). Moderately halophilic bacteria, which were the subject of the search, grow well in a salt range between 5 and 20 %. Therefore, the absorbance was compared with the estimated amount of ectoine (Fig. 2).

Most bacteria did not synthesise ectoine and hydroxyectoine or produced them in minimal quantities. Nevertheless, based on the number of mass detector counts, we identified 10 strains that were able to biosynthesise significant amounts of desirable products. Strains BHTA2, BHTA18, BHTA19, BHTA21, BHTA22, BHTA24, BHTA25 and BHTA30 synthesised mainly ectoine and negligible amounts of hydroxyectoine. Strain BHTA20 synthesised only ectoine, while BHTA8 synthesised mainly hydroxyectoine. Most of the selected microorganisms grew well under aerobic conditions in the medium containing a total of 10 % seawater. Only BHTA22 showed a low biomass concentration, so it was included in further studies. ANOVA analysis was carried out to validate the statistical differences observed between the strains. In all cases, the statistical significance of the results was confirmed and the p-value was less than 0.001.

All strains that produced significant amounts of ectoine and its derivative hydroxyectoine were isolated from the vicinity of Tyrawa spring. We identified them based on the 16S rDNA sequence and basic biochemical characteristics (Table 1).

**Table 1:** Identification of selected strains

Strain	Species name	GenBank accession number	Collection number
BHTA2	*Halobacillus* sp.	OR240871.1	KPD 1605*
BHTA8	*Bacillus velezensis*	OR240872.1	KPD 1606*
BHTA18	*Pseudalkalibacillus hwajinpoensis*	OR240874.1	KPD 1607*
BHTA19	*Virgibacillus salarius*	OR240982.1	B/00477**
BHTA20	*Salimicrobium halophilum*	OR240983.1	KPD 1608*
BHTA21	*Halobacillus halophilus*	OR240984.1	KPD 1609*
BHTA22	*Virgibacillus litoralis*	OR241005.1	KPD 1610*
BHTA24	*Thalassobacillus devorans*	OR241017.1	KPD 1611*
BHTA25	*Halobacillus sediminis*	OR241020.1	KPD 1612*
BHTA30	*Halobacillus sediminis*	OR241039.1	KPD 1604*

*Collection of Plasmids and Microorganisms, University of Gdańsk, Poland, **Polish Collection of Microorganisms

The results confirmed the differentiation of the isolated strains. Among the microorganisms, we identified six genera of bacteria. Four strains were classified as *Halobacillus* spp., two as *Virgibacillus* spp. and one each as *Bacillus* sp., *Pseudalkalibacillus* sp., *Salimicrobium* sp. and *Thalassobacillus* sp. In addition to the genetic identification, basic biochemical characteristics were also determined (Table 2).

**Table 2:** Determination of oxidase, l-alanine aminopeptidase and catalase activity, and the effect of temperature, pH and the different amounts of inorganic salts on the bacterial growth

Parameter	BHTA2	BHTA8	BHTA18	BHTA19	BHTA20	BHTA21	BHTA22	BHTA24	BHTA25	BHTA30
Temperature/°C	10-44 (37)	10-44 (37)	10-44 (37)	10-44 (30)	10-44 (30)	10-44 (30)	10-44 (30)	10-44 (30)	10-44 (30)	10-44 (37)
pH	6-9 (7)	6-9 (8)	5-9 (8)	3-9 (8)	3-9 (9)	6-9 (8)	3-8 (6-7)	6-9 (7-8)	5-9 (7-8)	6-9 (7-8)
Oxidase	+	+	-	+	+	+	+	-	+	+
l-alanine aminopeptidase	-	-	-	-	-	-	-	-	-	-
Catalase	+	+	+	+	+	+	+	+	+	+
Growth in medium without salt addition	-	+	+	+	-	-	-	+	-	-
*φ*(Instant Ocean (seawater))/%	5-20 (10-15)	5-15 (5)	5-25 (5)	5-25 (10-15)	5-15 (10)	5-20 (10)	5-15 (15)	5-20 (10)	10-25 (10)	5-25 (10-15)
(*m*(salt)/*V*(medium))/% NaCl	5-20 (5-10)	5-20 (5-10)	5-20 (5)	5-20 (5)	5-20 (5)	5-20 (15)	5-15 (10)	5-20 (5)	5 (5)	5 (5)
KCl	NG	5-25 (5-10)	5-20 (5)	5-25 (5-10)	5-20 (20)	10 (10)	5 (5)	5-15 (5)	NG	NG
(NH_4_)_2_SO_4_	NG	5-20 (5)	5-20 (5)	5-25 (10)	5-10 (10)	5 (5)	NG	5-25 (5)	NG	NG
MgSO_4_·7H_2_O	5-25 (5-10)	5-25 (5-10)	5-15 (5)	5-25 (5-10)	5-25 (5-10)	15-20 (15)	5-15 (5)	5-20 (5-10)	5-15 (15)	5-15 (5)

Assessment of enzymatic activity: - negative reaction, + positive reaction, NG=strain was not grown. Values in brackets are optimal

According to current knowledge, *Halobacillus halophilus* is the only ectoine producer previously described in detail (*18*). However, the final amount of ectoine synthesised by this species is low. Therefore, *H. halophilus* has not been industrially utilised for ectoine biosynthesis (*18*, *19*). There is a lack of knowledge about the cultivation conditions and the biosynthesis of ectoine by the other strains. Therefore, we decided to analyse the influence of temperature, pH and different salt amount on the bacteria (Table 2).

All bacteria showed catalase activity and lacked l-alanine aminopeptidase. Only *Pseudalkalibacillus hwajinpoensis* BHTA18 and *Thalassobacillus devorans* BHTA24 did not show oxidase activity. All strains grew in the entire analysed temperature range. However, the optimal value of this parameter varied depending on the strain. Most bacteria preferred temperatures around 30 °C, but *B. velezensis* BHTA8, *P. hwajinpoensis* BHTA18, *Halobacillus* sp. BHTA2 and *H. sediminis* BHTA30 had their optimum at 37 °C (Table 2). The pH range was strain dependent. Nevertheless, most of the tested microorganisms preferred slightly alkaline or neutral pH. This is not surprising since most halophiles normally live in such environments (*20*).

The growth of most halophilic microorganisms requires the presence of inorganic salt. High-salt medium is mandatory to stimulate ectoine biosynthesis. On the other hand, cell growth and excretion of the product can be limited by a high salt amount (*21*). Moreover, specific ions can affect ectoine metabolism in bacterial cells (*22*). There is no information about the media used for ectoine biosynthesis with the isolated strains. Therefore, to select the inorganic components of the media and determine their content range, the growth of the bacteria was tested in the presence of: NaCl, MgSO_4_, KCl and (NH_4_)_2_SO_4_ (Table 2).

Halophilic bacteria require the presence of sodium ions for their growth and metabolism, some are not able to grow in the absence of salt. Halophiles can be categorised into three primary groups based on their optimal salt preferences: mild (1–3 %), moderate (3–15 %) and extreme (15–30 %) (*23*). NaCl content, particularly sodium ions, is a known factor responsible for the regulation of ectoine biosynthesis. Ectoine production tends to show a positive correlation with increasing NaCl amount (*24*). The effect of NaCl is also observed at the molecular level. It influences the cell transport system and the expression of genes involved in the biosynthesis of ectoine (*ectABC*) and hydroxyectoine (*ectD*) (*25*). On the other hand, a high value of this factor limits the growth of bacteria.

In the case of KCl, it has been shown that some of the halophilic bacteria, *e.g*. *Halanaerobiales* sp. or *Salinibacter* sp., accumulate KCl to ensure osmotic balance in the cells and to stabilise the acid proteome. It is known that diaminobutyrate-2-oxoglutarate transaminase (*EctB*) is a pyridoxal phosphate (PLP)-dependent enzyme and K^+^ ions are necessary for its stability and activity (*26*, *27*). Ono *et al.* (*26*) reported that the presence of 0.01–0.5 M KCl increases the activity of *EctB* more than the presence of NaCl. Moreover, K^+^ is obligatory for the activity of many enzymes in the cells of extreme halophiles, which show a salt-dependent osmoregulation (*28*).

The ammonium ions can increase the expression of the *ectABC* gene cluster, which is involved in the synthesis of ectoine (*29*). It has also been assumed that ammonium sulphate increases the amount of l-aspartate-β-semialdehyde, which is the precursor of ectoine, and glutamate, which is responsible for the supply of co-substrates for *EctB* (*30*). These actions improve the production of ectoine by increasing the flow of substrate through the ectoine pathway.

Another factor that can influence the metabolism of halophilic bacteria is the presence of Mg^2+^. It has been shown that halophilic bacteria have a higher demand for this ion during their growth than non-halophilic microorganisms (*31*). It has also been suggested that high amounts of Mg^2+^ protect and stabilise macromolecules during dormancy. Moreover, the ratio between Mg^2+^ and Na^+^ varies depending on the physiological state and the presence of nutrients (*32*).

The results presented in Table 2 show that only *B. velezensis* BHTA8, *P. hwajinpoensis* BHTA18, *Virgibacillus salarius* BHTA19 and *T. devorans* BHTA24 were able to grow in a medium without added salt. The optimal NaCl content range is different for each strain. However, according to the nomenclature proposed by Kushner (*23*), most of the isolated strains can be classified as moderately halophilic bacteria, because they have an optimum in the range of 3–15 %. In the case of *Halobacillus sediminis* BHTA25 and BHTA30, there are some doubts and lower salt amounts should also be tested.

We observed no growth of *Halobacillus* sp. BHTA2, *H. sediminis* BHTA25 and BHTA30 in the presence of KCl only. Interestingly, the optimal value of this parameter reached 20 % for *S. halophilum* BHTA20. Growth in the presence of KCl depended significantly on the strain (Table 2). A similar effect was observed in the case of (NH_4_)_2_SO_4_. *Halobacillus* sp. BHTA2, *H. sediminis* BHTA25 and BHTA30 as well as *V. litoralis* BHTA22 did not grow. In other cases, the optimal amount of salt did not exceed 10 %. Most of the tested strains grew well in the presence of MgSO_4_·7H_2_O and had their optimum in the range of 5-10 % of the salt. The obtained results do not allow a simple determination of content limits. Future studies should consider synergistic and antagonistic effects between the ions. Therefore, artificial seawater (Instant Ocean) was used for further analysis. Artificial seawater is a mixture of different salts that mimics the natural environment, and it is easy to use in growth media. The isolated bacteria grow well when the seawater amount is up to 15 %. Additionally, *P. hwajinpoensis* BHTA18, *V. salarius* BHTA19, *H. sediminis* BHTA25 and BHTA30 were able to proliferate even in a 25 % solution (Table 2).

**Fig. 3:**
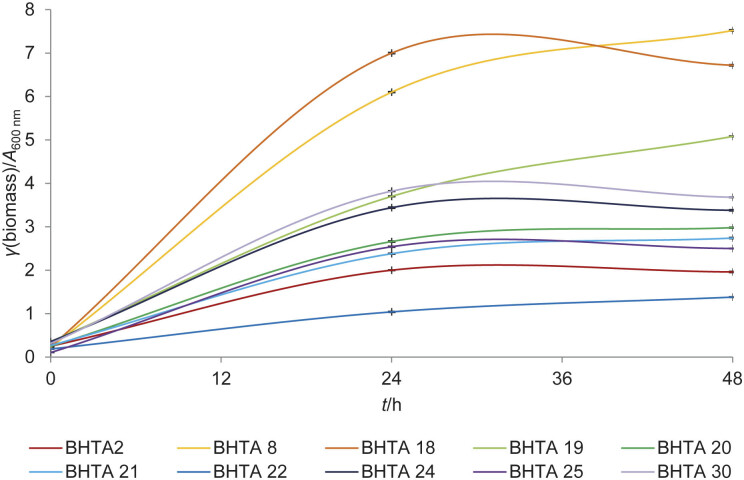
The growth of selected strains under optimal pH and temperature conditions

According to the presented results, we carried out biosynthesis of ectoine at optimal temperature and pH for each of the strains. We calculated the mean value for the optimal pH range, if the optimal value was defined as a range. The fermentation media contained 15 % Instant Ocean (seawater). Absorbance values were measured during cultivation (Fig. 3). Ectoine and hydroxyectoine were extracted after 48 h of fermentation and their concentration was quantified using HPLC (Table 3).

**Table 3:** Concentration of ectoine and hydroxyectoine determined by HPLC method

Strain	*γ*(ectoine)/(mg/L)	*γ*(hydroxyectoine)/(mg/L)
BHTA2	ND	ND
BHTA8	ND	ND
BHTA18	ND	ND
BHTA19	274±5	ND
BHTA20	4.5±0.3	ND
BHTA21	ND	ND
BHTA22	1.50±0.03	ND
BHTA24	ND	ND
BHTA25	5.30±0.03	ND
BHTA30	ND	ND

ND=not detected

All isolated strains grow well under the described conditions. The highest increase in absorbance was observed in the cultures *B. velezensis* BHTA8 and *P. hwajinpoensis* BHTA18, while the lowest value was observed in *Halobacillus* sp. BHTA2 and *V. litoralis* BHTA22. Ectoine concentration reached 274, 4.5, 1.5 and 5.3 mg/L in *V. salarius* BHTA19, *S. halophilum* BHTA20, *V. litoralis* BHTA22 and *H. sediminis* BHTA25, respectively. Hydroxyectoine was not detected. ANOVA analysis confirmed the statistical significance of the results and the p-value was less than 0.001.

Ectoine is produced on an industrial scale by *Halomonas elongata* DSM142 through a “bacterial milking” process. Using this microorganism Kunte *et al.* (*8*) obtained 7.4 g/L of ectoine, with a volumetric productivity of 0.22 g/L/h. The main disadvantage of the process was high salinity, up to 2.57 M, which can lead to corrosion of the equipment, increase production costs and inhibit the growth of the microorganisms. In addition, bacterial milking is effective only for Gram-negative bacteria. Therefore, other microorganisms were also considered as ectoine producers. At a much lower salt concentration, the biosynthesis of ectoine is carried out by *Brevibacterium epidermis* DSM20659. The strain produced 1.42 g/L of ectoine, with a productivity of 0.08 g/L/h (*33*). *Halomonas salina* BCRC17875 is another wild-type strain used for the biosynthesis of ectoine. In this case, ectoine production reached a concentration of 13.96 g/L after 44 h of cultivation and a NaCl concentration of 2 M (*34*). High concentrations of ectoine can be achieved with the use of genetically modified microorganisms. *Escherichia* coli and *Corynebacterium glutamicum* are commonly used for this purpose. The genetically modified *E. coli* BL21 strain yielded 60.7 g/L with a volumetric productivity of 1.08 g/L/h using glucose as the sole carbon source under low salt concentration conditions (*35*). The metabolically engineered *C. glutamicum* produced significant amounts of ectoine in low-salt environments. *C. glutamicum* Ect10 showed the ability to synthesise 115.87 g/L of ectoine, making it one of the most efficient ectoine producers documented so far (*36*).

The biosynthesis of hydroxyectoine is closely related to the presence of *ectD* gene, which encodes ectoine hydroxylase. Hydroxyectoine impedes the purification of ectoine and reduces its biosynthesis yield, so strains with high ectoine hydroxylase activity require a genetic modification to inhibit this function (*37*, *38*). The presence of the *ectD* gene in *V. salarius* has been confirmed (*39*). *V. salarius* BHTA19 synthesised hydroxyectoine, as shown by LC-MS analysis (Fig. 2), but it was not detected by HPLC (Table 3). This suggests that the expression of the *ectD* gene and the activity of ectoine hydrolase depend on the culture conditions and require further investigation.

Among the isolated strains, *V. salarius* BHTA19 showed the most promising properties. The strain not only produced the highest concentration of ectoine, but it also showed broad tolerance to the type of salt and its amount as well as temperature and pH. There is a lack of knowledge about the cultivation conditions and the biosynthesis of ectoine by *V. salarius*. However, *Virgibacillus* sp. is already known as an ectoine producer. To our knowledge, only a few genera have been described so far. *Virgibacillus pantothenticus*, *Virgibacillus halodenitrificans* and *Virgibacillus salarius* 19.PP.SC1.6 are worth mentioning (*25*, *39*, *40*). Unfortunately, their production efficiency is not impressive. Considering that *V. salarius* BHTA19 was cultivated in a flask culture, the obtained amount of ectoine seems to be promising. For this reason, further studies are required to determine the potential of the strain for industrial application.

## CONCLUSIONS

Ectoine is one of the most marketable biotechnological products. There is a consistent increase in the number of its applications, leading to a growing demand for this metabolite. Although ectoine is produced on an industrial scale, the process still needs to be improved. The high salt amounts used in current methods require the use of the equipment that is suitably resistant. Furthermore, managing production waste that contains significant quantities of salt is a challenge. Ongoing research efforts should focus not only on the improvement of efficiency and the reduction of production costs, but also on the identification of natural ectoine-producing strains that can perform biosynthesis at low or moderate salt amounts.

As a result of the presented research, a moderate halophile *Virgibacillus salarius* BHTA19 has been identified as a new potential producer of ectoine. This study showed that after 24 h of shake flask culture, the concentration of ectoine reached 274 mg/L. The concentration of ectoine is lower than currently reported for other microorganisms, but BHTA19 was not optimised, so the results cannot be directly compared. Nevertheless, the presented research provides a solid basis to encourage the development of an innovative technology.
